# N^6^-methyladenosine-modified circ_104797 sustains cisplatin resistance in bladder cancer through acting as RNA sponges

**DOI:** 10.1186/s11658-024-00543-3

**Published:** 2024-02-23

**Authors:** Congjie Xu, Jiaquan Zhou, Xiaoting Zhang, Xinli Kang, Shuan Liu, Mi Song, Cheng Chang, Youtu Lin, Yang Wang

**Affiliations:** 1 Department of Urology, Hainan General Hospital (Hainan Affiliated Hospital of Hainan Medical University), Haikou, Hainan People’s Republic of China; 2 Shenzhen Baoan District Songgang People’s Hospital, Shenzhen, Guangdong People’s Republic of China; 3 Department of Urology, The Third People’s Hospital of Danzhou, Danzhou, Hainan People’s Republic of China

**Keywords:** Bladder cancer, Cisplatin resistance, circ_104797, miR-103a, N^6^-methyladenosine

## Abstract

**Background:**

Bladder cancer (BCa) ranks among the predominant malignancies affecting the urinary system. Cisplatin (CDDP) remains a cornerstone therapeutic agent for BCa management. Recent insights suggest pivotal roles of circular RNA (circRNA) and N^6^-methyladenosine (m6A) in modulating CDDP resistance in BCa, emphasizing the importance of elucidating these pathways to optimize cisplatin-based treatments.

**Methods:**

Comprehensive bioinformatics assessments were undertaken to discern circ_104797 expression patterns, its specific interaction domains, and m^6^A motifs. These findings were subsequently corroborated through experimental validations. To ascertain the functional implications of circ_104797 in BCa metastasis, in vivo assays employing CRISPR/dCas13b-ALKBH5 were conducted. Techniques, such as RNA immunoprecipitation, biotin pull-down, RNA pull-down, luciferase reporter assays, and western blotting, were employed to delineate the underlying molecular intricacies.

**Results:**

Our investigations revealed an elevated expression of circ_104797 in CDDP-resistant BCa cells, underscoring its pivotal role in sustaining cisplatin resistance. Remarkably, demethylation of circ_104797 markedly augmented the potency of cisplatin-mediated apoptosis. The amplification of circ_104797 in CDDP-resistant cells was attributed to enhanced RNA stability, stemming from an augmented m6A level at a distinct adenosine within circ_104797. Delving deeper, we discerned that circ_104797 functioned as a microRNA reservoir, specifically sequestering miR-103a and miR-660-3p, thereby potentiating cisplatin resistance.

**Conclusions:**

Our findings unveil a previously uncharted mechanism underpinning cisplatin resistance and advocate the potential therapeutic targeting of circ_104797 in cisplatin-administered patients with BCa, offering a promising avenue for advanced BCa management.

## Background

Bladder cancer is a leading malignancy of the urinary system, ranking among the most common global tumors. In the Chinese demographic, it is the primary urogenital malignancy, with incidence rates in males being three to four times higher than in females [1–3]. A majority, approximately 90%, of these cases are identified as urothelial carcinomas, commonly known as transitional cell carcinoma. Subsequently, squamous cell carcinoma and adenocarcinoma represent 3–7% and 2% of cases, respectively. It is noteworthy that 70–85% of transitional cell carcinomas are categorized as non-muscle invasive bladder cancer (NMIBC). Postsurgical observations indicate that 10–15% of NMIBC cases evolve into muscle-invasive stages. The outlook for these patients is concerning as almost half do not survive beyond 5 years post-diagnosis [4]. The primary clinical intervention for NMIBC is transurethral resection of the bladder tumor (TURBT). Yet, post-TURBT data suggests a recurrence rate of 50–80%, with 10–25% of these recurrences progressing to muscle-invasive stages [5–7]. The main challenges in bladder cancer treatment are postsurgical recurrence and the heightened malignancy following recurrence. To address these challenges, intravesical chemotherapy and immunotherapy have become standard postoperative treatments for NMIBC patients [8]. Common chemotherapy protocols include methotrexate + vinblastine + doxorubicin + cisplatin (M-VAP), gemcitabine + cisplatin (GC), and methotrexate + vinblastine + cisplatin (MVP), with efficacy rates spanning 40–65% [9]. Cisplatin, a widely used chemotherapeutic agent for various cancers, including bladder cancer, often encounters challenges in treating urothelial carcinoma owing to diverse pathological classifications, genetic differences, and drug resistance. The primary obstacle remains the unclear molecular pathways that lead to resistance against cisplatin.

Circular RNA (circRNA) represents a distinct category of RNA molecules, distinguished by their closed-loop configuration, discovered nearly four decades ago. Initially, they were considered accidental outcomes of splicing anomalies, with early hypotheses questioning their biological significance [10]. Contrasting with traditional linear non-coding RNAs, circRNAs are products of precursor mRNA (pre-mRNA) back-splicing. This mechanism entails the fusion of the 5′ terminus of an exon or intron with the 3′ terminus of a different exon or intron, leading to the genesis of a singular covalently sealed RNA loop [11–14]. Recent investigations highlight the impressive cellular preservation of circRNAs, their notable prevalence, and their intrinsic cell and tissue selectivity. Additionally, circRNAs exhibit distinct expression trends throughout diverse developmental stages [15, 16].

In the realm of oncology, circRNAs have been identified as crucial molecular entities, particularly in their capacity as miRNA sponges, a role that has gained significant attention in tumor biology [17]. For example, circMYLK, acting as a pro-oncogenic molecule, engages competitively with miR-29a, thereby modulating vascular endothelial growth factor A (VEGFA)/vascular endothelial growth factor receptor (VEGFR) expression and the subsequent Ras/ERK signaling pathway. This interaction promotes epithelial–mesenchymal transition (EMT), enhancing bladder cancer cell activities such as proliferation, migration, angiogenesis, and cytoskeletal alterations [18]. Similarly, circHIPK3, through its competitive interaction with miR-124, regulates its activity, impacting various cellular functions [19]. circPVT1, by engaging with the endogenous miR-125 family, boosts cell growth, suggesting its potential as a diagnostic indicator for gastric cancer [20]. However, some studies indicate that many circRNAs might not possess miRNA binding potential [21], emphasizing the need for a comprehensive understanding of their multifarious regulatory functions.

In the context of oncology, the significance of circRNA in modulating resistance to cisplatin, a cell cycle-independent anti-cancer agent, is increasingly recognized. Cisplatin has a broad anti-cancer spectrum and is particularly effective against hypoxic cells. Once internalized, cisplatin rapidly dissociates and forms hydrated cations that bind to cellular DNA, thereby disrupting the DNA double helix and its functional integrity [22, 23]. The agent is widely employed in the treatment of various malignancies, including lung, prostate, and bladder cancers, as well as malignant lymphoma and squamous cell carcinoma. Specifically, circ-Cdrlas enhances cisplatin susceptibility in bladder cancer cells by inhibiting miR-1270, which in turn upregulates apoptotic peptidase activating factor 1 (APAF1) expression [24]. Targeted reduction of circELP3 levels using siRNA significantly attenuates bladder cancer cell proliferation and cisplatin resistance in vitro, while also promoting apoptosis. Moreover, circELP3 interference inhibits tumor xenograft expansion in animal models and is strongly associated with advanced tumor stages and lymph node metastasis [25]. In hepatocellular carcinoma, reduced levels of circ-101505 are linked to poor survival and increased cisplatin resistance. Overexpression of circ-101505 not only inhibits cancer cell growth but also enhances cisplatin toxicity by sponging miR-103 and upregulating neuron-derived orphan receptor 1 (NOR1) expression [26].

Remarkably, a wealth of research has established the critical importance of m^6^A in both the metabolic processes and functional aspects of circRNAs. The influence of m^6^A on circRNAs primarily manifests in four distinct areas: The role of m^6^A in circRNA biogenesis, as shown in studies such as that of Timoteo et al. on circZNF609 [27]; the importance of m^6^A in the cytoplasmic export of circRNAs, exemplified by circNSUN2 [28]; the involvement of m^6^A in circRNA degradation through mechanisms, such as the YTHDF2-HRSP12-RNase P/MRP axis [29]; and the role of m^6^A in circRNA translation, facilitated by factors like YTHDF3 and eIF4G2 [30].

In the present study, we utilized circRNA sequencing data obtained from clinical samples of bladder cancer and corroborated these findings through experimental validation in cisplatin-resistant bladder cancer cell lines. We identified circ_104797 as a key molecule of interest. Our data reveal that the stability of circ_104797 is influenced by m^6^A methylation modifications. Additionally, circ_104797 competitively interacts with downstream miR-660-3p and miR-103a, thereby exacerbating cisplatin resistance in bladder cancer cells. This research aims to elucidate the roles and underlying mechanisms by which m^6^A modifications regulate circ_104797, thereby contributing to cisplatin resistance in bladder cancer. These insights could potentially open new avenues for research and therapeutic interventions in the management of cisplatin-resistant bladder cancer.

## Methods

### Patients and tissue specimen collection

The research protocol was conducted in alignment with the principles of the Declaration of Helsinki. Prior to the commencement of the study, written consents were secured from all participating patients. Tissue samples, both malignant and adjacent normal, were procured from patients with bladder cancer (BCa) undergoing surgical procedures. Comprehensive follow-ups were conducted for all patients, with overall survival (OS) being calculated from the surgery date to either the date of demise or the last recorded follow-up for those still alive.

### Cell culture and transfection

In our research, we utilized a collection of BCa cell lines, namely T24 and BIU-87, with SVHUC-1 cells serving as the standard control. These cell lines were sourced from the American Type Culture Collection (ATCC, Manassas, VA, USA) and the National Infrastructure of Cell Line Resource in China. Standard culture conditions for the BCa cell lines involved Roswell Park Memorial Institute (RPMI) 1640 or Dulbecco’s Modified Eagle Medium (DMEM) mediums enriched with 10% fetal bovine serum (sourced from Invitrogen, Carlsbad, CA, USA). These cells were incubated at 37 °C in an environment with 5% CO_2_. For transfection procedures, we employed lipo3000 (from Invitrogen) in adherence to the guidelines provided by the manufacturer, ensuring a consistent plasmid quantity of 1 μg for our experimental protocols.

To develop the cisplatin-resistant bladder cancer cell lines, we employed a gradual adaptation approach. Initially, the T24 and 5637 bladder cancer cell lines were exposed to a sub-lethal concentration of cisplatin. This initial dose was carefully chosen to be just below the level that induces significant cell death, ensuring the survival of a small population of less sensitive cells. Over the course of 6 months, we incrementally increased the cisplatin concentration after each cell adaptation and recovery phase. This method allowed for the selection and proliferation of cells with heightened resistance to cisplatin.

### circRNA microarray

From the Cancer Center, we procured samples comprising four BCa specimens and their corresponding adjacent normal tissues. The protocols outlined by Arraystar (Rockville, MD, USA) guided our sample preparation and microarray hybridization processes. To isolate circular RNAs, linear RNAs were eliminated using RNase R digestion (sourced from Epicentre Technologies, Madison, WI, USA). These circRNAs were then transcribed into fluorescent circRNA using the Arraystar Super RNA Labeling Kit. The fluorescently labeled circRNAs were subsequently hybridized to the Arraystar Human circRNA Array V2 (8 × 15 K, Arraystar) and the results were captured using the Agilent Scanner G2505C (Jamul, CA, USA). To identify circRNAs with significant differential expression (with an |average normalized fold change| of ≥ 1.3) between the groups, we employed fold change thresholds.

### RNA quantitative real-time polymerase chain reaction

RNA was isolated utilizing the TRIzol reagent (sourced from Invitrogen, Carlsbad, USA). From the extracted RNA, cDNA was synthesized using two micrograms as the starting material. Subsequently, 1 µl of this cDNA (equivalent to 0.2 µg) was employed for polymerase chain reaction (PCR) amplification. The real-time polymerase chain reaction (RT-PCR) was executed using SYBR Green SuperMix (obtained from Roche, Basel, Switzerland) and the ABI7900HT Fast Real-Time PCR system (from Applied Biosystems, CA, USA), with 1 µl cDNA serving as the template. For normalization, β-actin or U3 was utilized as an internal reference.

### Actinomycin D and RNase R treatment

BCa cells were seeded into six-well plates and, after 24 h, reached approximately 60% confluency. Subsequently, the cells were exposed to either 5 μg/ml actinomycin D or dimethyl sulfoxide (DMSO) and harvested at specified intervals. For RNA processing, 2 μg of total RNA was treated with 3 U/μg of RNase R (sourced from Epicentre Technologies, Madison, WI, USA) and incubated for 15 min at 37 °C. Post actinomycin D or RNase R treatment, the expression levels of circ_104797 and associated mRNAs were assessed using quantitative RT-PCR (qRT-PCR).

### Nuclear and cytoplasmic extraction

Cytoplasmic and nuclear components were separated using the PARIS™ Kit (AM1556, Thermo Fisher Scientific, Waltham, USA) as per the manufacturer’s guidelines. In brief, BCa cells were subjected to lysis in cell fraction buffer and incubated on ice for 10 min. Post incubation, the mixture was centrifuged at 500 × g for 3 min at 4 °C and the resulting supernatant, representing the cytoplasmic fraction, was carefully collected. The remaining pellet was rinsed with cell fraction buffer to obtain the nuclear fraction.

### RNA fluorescence in situ hybridization (FISH)

The probe sequence specific to circ_104797 was procured from Sangon Biotech (Shanghai, China). Cells, once fixed, were rinsed with phosphate-buffered saline (PBS) and subsequently treated with RNase R at 37 °C for a duration of 15 min, followed by a second fixation. The cell mixture was then dispensed onto sterilized glass slides and dehydrated sequentially using 70%, 80%, and 100% ethanol. Hybridization ensued in a humidified, dark chamber at 37 °C overnight. Post-hybridization, the slides were rinsed twice with a solution of 50% formamide/2 × SSC for 5 min each. Subsequently, the slides were treated with reagents from the Alexa FluorTM 488 Tyramide SuperBoost™ Kit (Thermo Fisher Scientific, Waltham, USA) for 30 min and then sealed using parafilm embedded with 4′,6-diamidino-2-phenylindole (DAPI). Fluorescent images were captured using a fluorescence microscope. ImageJ software was employed for fluorescence intensity analysis, while the OLYMPUS FV1000 software was utilized to assess Pearson’s correlation coefficient.

### Methylated RNA immunoprecipitation PCR (MeRIP-qPCR)

To quantitatively assess m^6^A-modified mRNA, MeRIP-qPCR was employed. Post mRNA extraction, the anti-m^6^A antibody and anti-IgG (sourced from Cell Signaling Technology) were bound to protein A/G magnetic beads in immunoprecipitation (IP) buffer, which contained 140 mM NaCl, 20 mM Tris (pH 7.5), 2 mM EDTA, and 1% NP-40 along with RNase and protease inhibitors. This mixture was incubated overnight at 4 °C. Subsequent to this incubation, the RNA-bead complex was separated using elution buffer. The final step involved determining RNA enrichment via qRT-PCR, where the enrichment was calculated using the 2-ΔΔCt method, comparing the eluate to the input sample.

### RNA-binding protein immunoprecipitation (RIP)

The RIP assay was conducted using the Magna RIP RNA-Binding Protein Immunoprecipitation Kit (Millipore) as per the manufacturer’s guidelines. In essence, BCa cells, post 48-h transfection, were collected and subjected to lysis in RIP lysis buffer, with incubation on ice for 30 min. Following centrifugation, the resultant supernatant was combined with 30 μl of Protein-A/G agarose beads (sourced from Roche, USA) and specific antibodies. This mixture was incubated overnight. Subsequently, the immune complexes underwent centrifugation and were washed six times using a washing buffer. Protein analysis from the bead-bound complexes was carried out via western blotting, while the RNA extracted from the immunoprecipitation was evaluated using qRT-PCR.

### RNA pull-down

Using streptavidin-coated magnetic beads (Invitrogen, Carlsbad, USA), the biotinylated RNA complex was isolated from cell lysates as per the manufacturer’s guidelines. The enrichment of circRNA in the isolated fractions was determined using qRT-PCR. Proteins bound to the beads were subsequently eluted and subjected to sodium dodecyl-sulfate polyacrylamide gel electrophoresis (SDS-PAGE). Western blotting was employed to identify and analyze the proteins present in the captured complex.

### Luciferase reporter assay

The circRNA sequence was integrated into the p-GLO Dual-Luciferase vector (Vigenebio, Maryland, USA), with specific mutations introduced at the binding sites. BCa cells, seeded in 24-well plates at 30% confluency, were co-transfected 24 h later with 800 ng of the p-GLO Dual-Luciferase reporter and 800 ng of vectors. After an incubation period of 48 h, the dual luciferase reporter assay system (Promega, Madison, WI) was employed to determine the relative luciferase activity, which was expressed as the Firefly to Renilla luciferase activity ratio. The Renilla luciferase activity served as an internal normalization control. For the ALKBH5 overexpression samples, the Luc/Rluc ratio was further standardized to the control group’s value.

### Cell proliferation assay

Cellular proliferation was evaluated using the CCK-8 assay kit (Transgen, China). Cells were plated in 96-well plates at a concentration of 3 × 10^3^ cells per well. Post-seeding, cells were incubated for specific durations:12, 24, 48, and 72 h. After each interval, CCK-8 reagent was added to the wells, and the plates were incubated at 37 °C for an additional 3 h. The optical density of each well was subsequently recorded at 450 nm using a microplate spectrophotometer.

### Wound healing migration assays

Wound healing assays were performed using a standard protocol. Cells were seeded in six-well chamber slides at a concentration of 5 × 10^5^ cells/well. A sterile 10 μl pipette tip was employed to create a linear scratch in the cell monolayer. After a 48-h incubation period, images were taken to assess the migration of cells into the wound area. The percentage of wound closure was calculated using the formula: wound closure (%) = [(initial wound width − width after 48 h)/initial wound width] × 100%.

### Cell apoptosis assay

In a 12-well plate, cells transfected with the designated vector were seeded at a concentration of 2 × 10^5^ cells per well, ensuring 70–80% confluency. Following a 48-h incubation, apoptosis was evaluated using the caspase-3 ELISA assay kit (Hcusabio, China). Given its role in mediating DNA and cytoskeletal protein degradation, caspase-3 is a critical marker for apoptotic pathways and inflammatory responses. Each experimental setup was performed in triplicate to ensure consistency and precision.

### Xenograft model

In this research, we incorporated female BALB/c nude mice aged between 4 and 5 weeks. These mice were divided into two distinct groups, each consisting of six individuals. The living conditions for these mice were maintained at specific-pathogen-free standards in line with conventional animal housing protocols. We administered a subcutaneous injection of 5 million transfected bladder cancer cells into the right flank region of each mouse. To track the progression of the tumors, we measured their volumes on a weekly basis. At 4 weeks following the injection, the mice were humanely euthanized under anesthesia, and the tumors were extracted from the subcutaneous tissues. These excised tumors were then analyzed for both their volume and weight. Moreover, our methods for handling and experimenting with the animals were in strict compliance with the ethical guidelines for animal research as set by our institution.

### Bioinformatic analysis

To investigate the potential biological functions of circ_104797, we performed a prediction by SRAMP (http://www.cuilab.cn/sramp) and RMBase v2.0. CircRNA-miRNA interactions were predicted using Arraystar’s miRNA target prediction software based on TargetScan and miRanda.

### Statistical analysis

Data are expressed as mean ± standard deviation (SD). Statistical evaluations were conducted using GraphPad Prism 8.0 (GraphPad, San Diego, CA, USA) and SPSS version 20.0 (IBM, SPSS, Chicago, IL, USA). For pairwise comparisons, either a two-sided Student’s *t*-test or a two-tailed Mann–Whitney *U*-test was applied, contingent on the data distribution. For multiple group comparisons, one-way analysis of variance (ANOVA) was employed, followed by Bonferroni post hoc tests for further analysis. A *P*-value less than 0.05 was deemed statistically significant. In figures or accompanying legends, significance levels are indicated as *P < 0.05 and **P < 0.01.

## Results

### circ_104797 was an unfavorable circRNA for bladder cancer

**Fig. 1:**
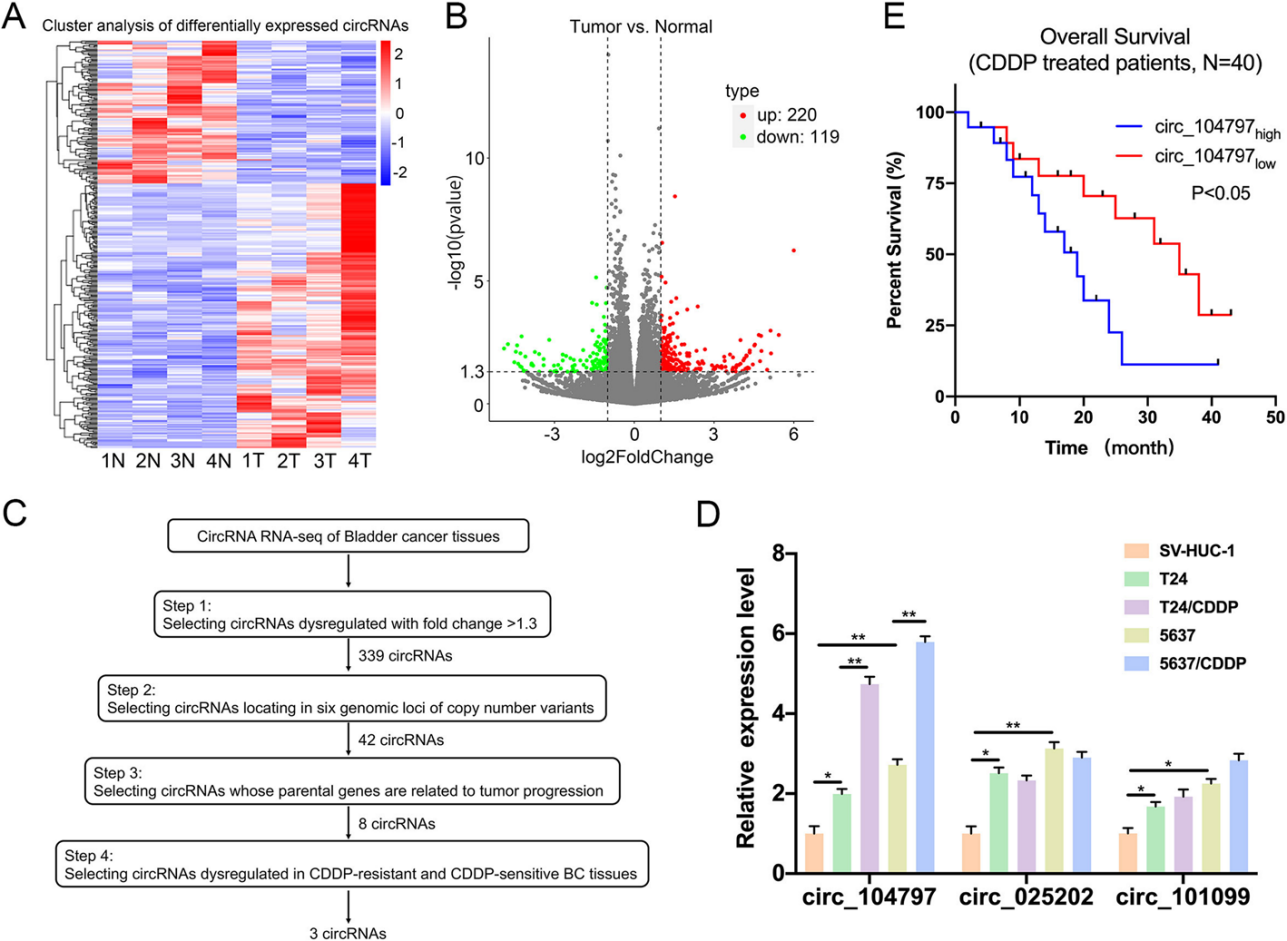
circRNA_104797 is upregulated in BCa. **A** Heatmap illustrating the differential expression profiles of circRNAs between four matched sets of bladder cancer and adjacent normal tissues; **B** volcano plot representing the results of circRNA microarray analysis; **C** schematic diagram outlining the selection criteria for identifying candidate regulatory circRNAs enriched in bladder cancer; **D** comparative expression levels of circRNA_104797 across SV-HVC-1, T24, T24/CDDP, 5637, and 5637/CDDP cell lines; **E** Kaplan–Meier survival analysis depicting overall survival rates in bladder cancer patients stratified by low or high expression levels of circRNA_104797 (*n* = 40)

In this investigation, we employed a high-throughput human circRNA microarray to analyze carcinoma and adjacent non-tumorous tissues from four patients with bladder cancer (BCa). As depicted in Fig. 1A and B, both the cluster map and scatterplot revealed differential expression of 339 circRNAs in the BCa group relative to the control: 220 were upregulated, while 119 were downregulated. From this pool of differentially expressed circRNAs, and considering a fold change exceeding 1.3, circRNA features, and their parental genes, we pinpointed three circRNAs (circ_104797, circ_025202, and circ_101099) for further validation and detailed analysis (Fig. 1C). The expression profiles of these circRNAs in standard SV-HUC-1 cells versus BCa cells (T24 and 5637) were assessed using quantitative real-time PCR (qRT-PCR). The data indicated an elevated expression of these circRNAs in BCa cells relative to the standard cells, with circ_104797 showing the most pronounced upregulation (Fig. 1D). Furthermore, circ_104797 levels were observed to be higher in cisplatin-resistant BCa cell lines (T24/CDDP and 5637/CDDP) compared with their primary BCa counterparts (T24 and 5637; Fig. 1D). Additionally, patients with BCa with elevated circ_104797 expression demonstrated a reduced overall survival rate compared with those with lower expression levels (Fig. 1E). These findings imply a potential role of circ_104797 in cisplatin resistance in BCa and its correlation with unfavorable clinical outcomes.

### Characterization of circ_104797 in CDDP-resistant BCa cells

**Fig. 2:**
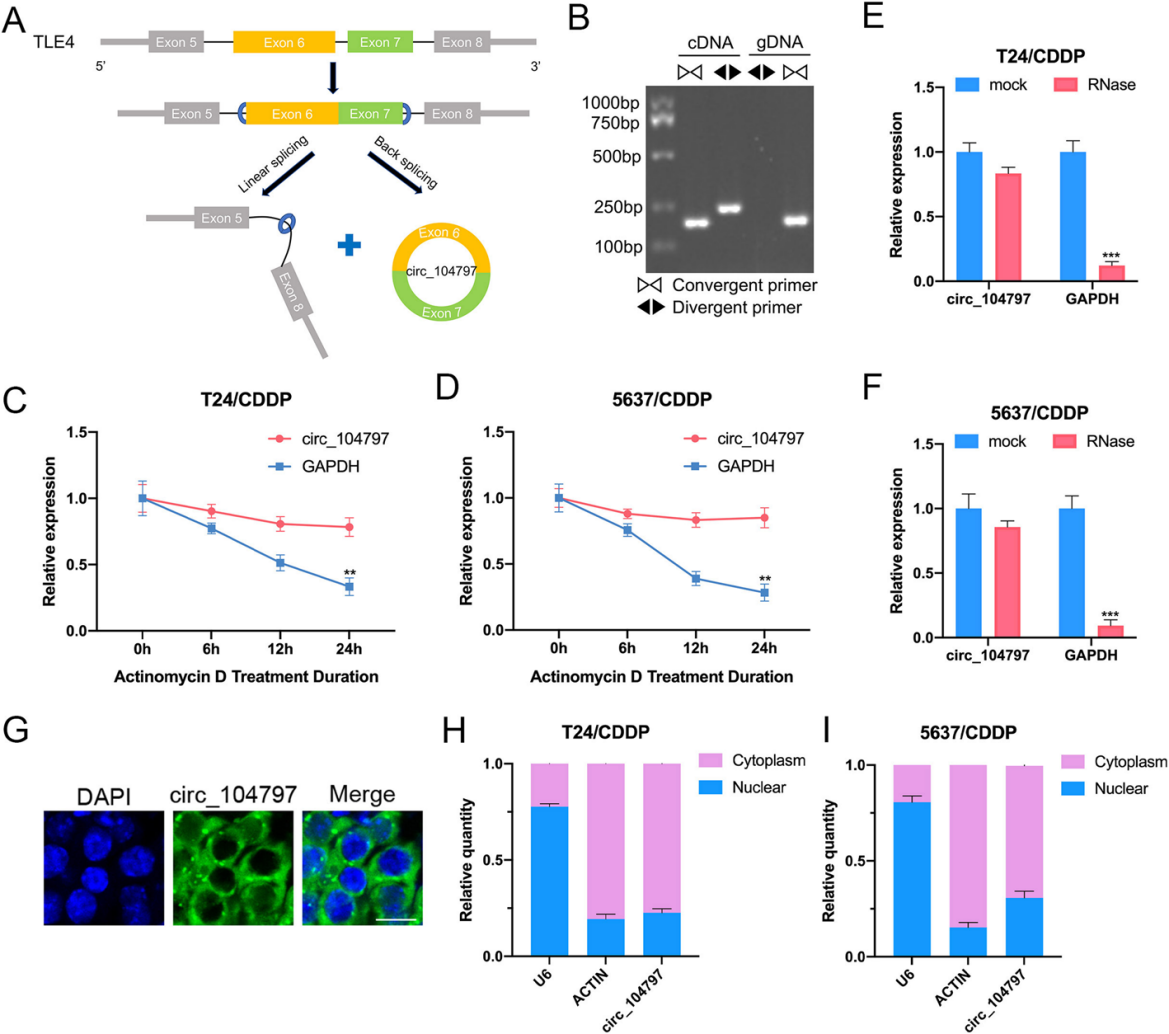
The characteristics of circ_104797. **A** Diagrammatic representation depicting the formation of circ_104797; **B** gel electrophoresis demonstrates amplification of circ_104797 using divergent primers from total cDNA, but not from genomic DNA (gDNA); **C**, **D** RT-qPCR analysis indicating the relative abundance of GAPDH mRNA and circ_104797 in T24/CDDP (**C**) and 5637/CDDP (**D**) cells post actinomycin D (Act-D) treatment at specified time intervals; **E**, **F** RT-qPCR results confirm the resistance of circ_104797 to RNase R digestion in T24/CDDP (**E**) and 5637/CDDP (**F**) cells; **G** subcellular localization of circ_104797 in BCa cells as determined by FISH assay; **H**, **I** RT-qPCR assessment of circ_104797, actin, and U6 distribution in the cytoplasmic and nuclear fractions of T24/CDDP (**H**) and 5637/CDDP (**I**) cells

Our analysis revealed that circ_104797 originates from the exonic regions 6 and 7 of the TLE4 gene (Fig. 2A). RT-PCR assays, utilizing divergent primers, confirmed the amplification of circ_104797 in cDNA synthesized from total RNA, but not from genomic DNA (Fig. 2B). Upon actinomycin D exposure, circ_104797 displayed enhanced stability in contrast to GAPDH mRNA in CDDP-resistant BCa cells (Fig. 2C, D). To further validate the circular nature of circ_104797, we employed RNase R treatment. Notably, circ_104797 remained largely resistant to RNase R-mediated degradation, while the linear counterpart, GAPDH mRNA, was predominantly degraded in CDDP-resistant BCa cells (Fig. 2E, F). Subsequent investigations using FISH assays and nuclear-cytoplasmic fractionation techniques ascertained the presence of circ_104797 in both the cytoplasmic and nuclear compartments of CDDP-resistant BCa cells (Fig. 2G–I). Collectively, these findings underscore the circular configuration and intracellular distribution of circ_104797 in CDDP-resistant BCa cells.

### circRNA_104797 is modulated by m^6^A RNA methylation

**Fig. 3:**
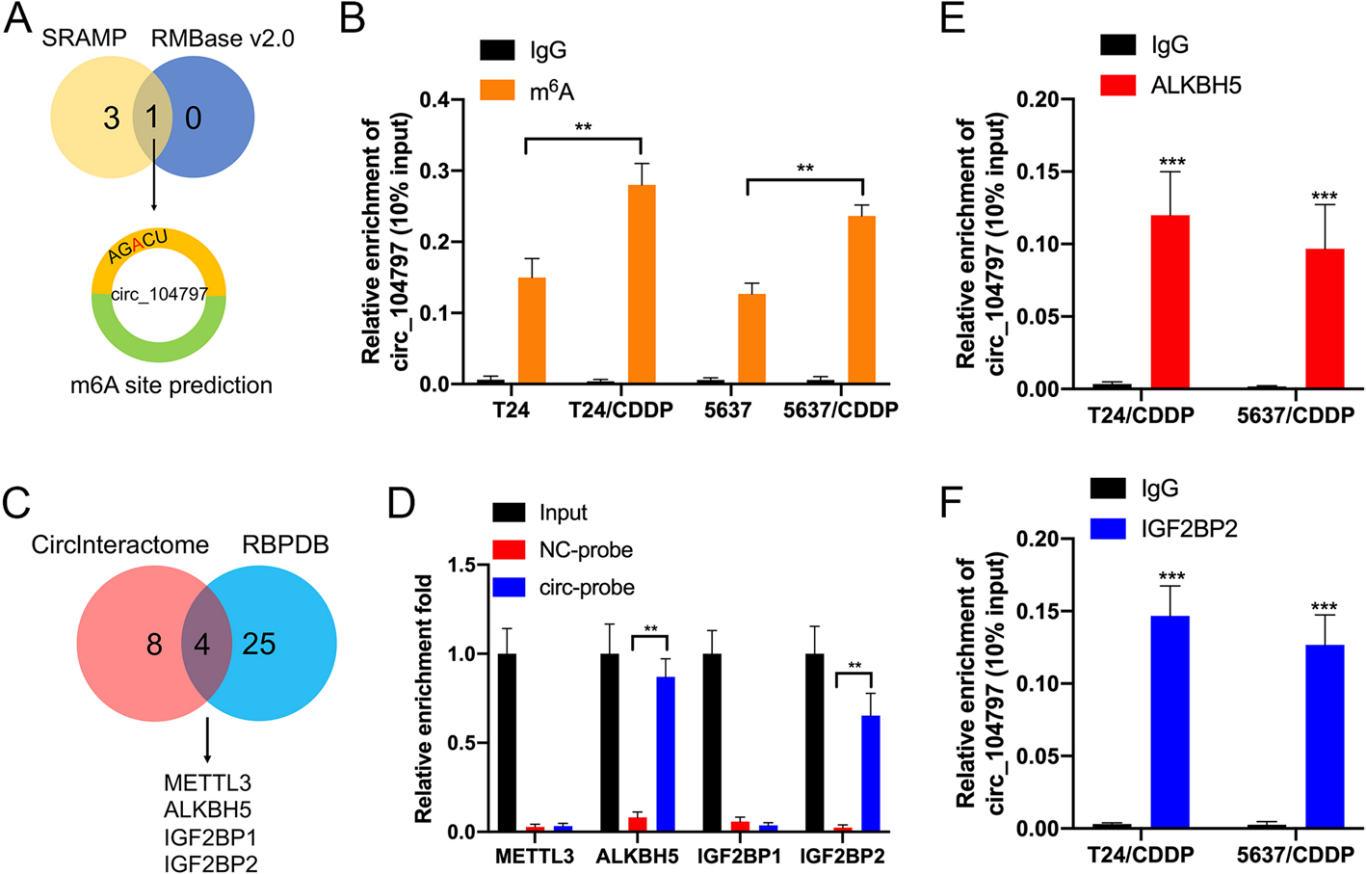
circ_104797 is modulated by m^6^A RNA methylation. **A** Identification of the potential m^6^A site in circ_104797, as determined by the convergence of results from the sequence-based N^6^-methyladenosine (m^6^A) site prediction tools SRAMP and RMBase v2.0; **B** MeRIP-qPCR analysis illustrating the relative enrichment of circ_104797 when immunoprecipitated with m^6^A antibody (m^6^A-IP) compared with IgG in BCa cells; **C** bioinformatics analysis using online platforms (https://circinteractome.nia.nih.gov/, http://rbpdb.ccbr.utoronto.ca/) suggested potential interactions between circ_104797 and specific RNA binding proteins (RBP); **D** RNA pulldown assays complemented by western blotting revealed interactions of circ_104797 with METTL3, ALKBH5, and IGF2BP1/2; **E**, **F** RBP immunoprecipitation (RIP) followed by qPCR analysis indicated significant enrichment of circ_104797 in the anti-ALKBH5 and anti-IGF2BP2 antibody groups compared to the IgG control in T24/CDDP (**E**) and 5637/CDDP (**F**) cells

circRNA_104797 appears to be produced from the back-splicing of the 6th and 7th exons of the TLE4 gene. Yet, the precise regulatory mechanisms governing its expression are not fully understood. m^6^A, a predominant RNA base modification, has been implicated in circRNA biogenesis [28, 30]. Specifically, circRNAs containing m^6^A modifications are believed to undergo cleavage by the YTHDF2-HRSP12-RNase P/MRP complex [31]. This modification typically resides within the ‘RRm^6^ACH’ consensus sequence (R = G or A; H = A, C, or U) [32]. Utilizing SRAMP and RMBase v2.0 for m^6^A site prediction, we identified a potential m^6^A site within circRNA_104797, proximal to its junction region (Fig. 3A). Notably, m^6^A-specific immunoprecipitation (MeRIP-qPCR) revealed elevated m^6^A levels in circRNA_104797 from CDDP-resistant BCa cells relative to their wild-type counterparts (Fig. 3B). This suggests a potential regulatory role of m^6^A modification in circRNA_104797 expression during cisplatin resistance development. Bioinformatic analyses indicated potential interactions between circ_104797 and RNA binding proteins (RBPs), such as METTL3, ALKBH5, IGF2BP1, and IGF2BP2 (referenced from circinteractome.nia.nih.gov and rbpdb.ccbr.utoronto.ca; Fig. 3C). RNA pull-down assays targeting circ_104797 confirmed significant interactions with ALKBH5 and IGF2BP2 (Fig. 3D). Moreover, RBP immunoprecipitation (RIP) assays revealed enrichment of circ_104797 in both the anti-ALKBH5 (Fig. 3E) and anti-IGF2BP2 (Fig. 3F) antibody fractions, underscoring the molecular associations between circ_104797 and the RBPs ALKBH5 and IGF2BP2.

### ALKBH5 inhibited the m^6^A modification process of circ_104797

**Fig. 4:**
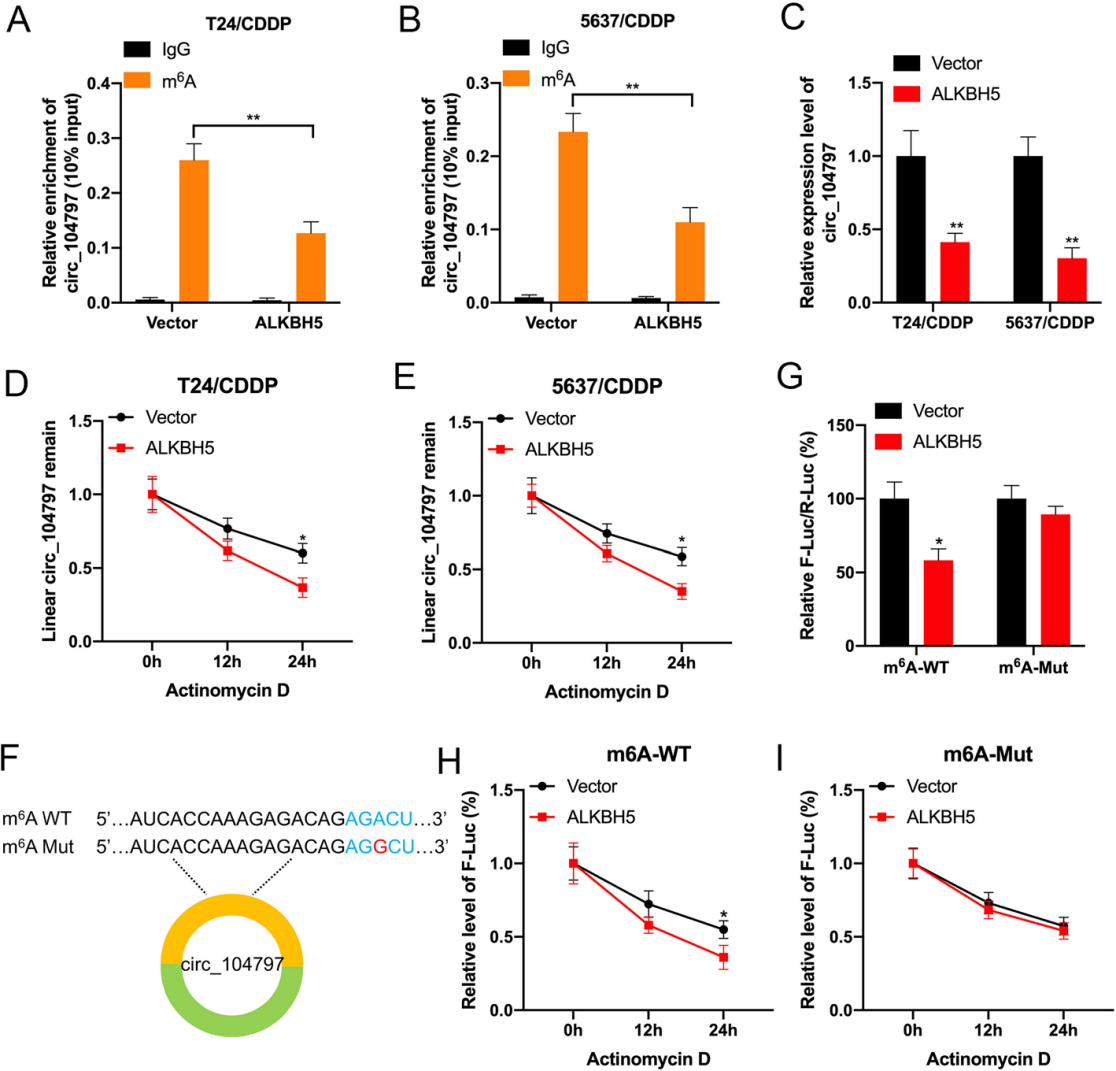
ALKBH5 inhibited the m^6^A modification process of circ_104797. **A**, **B** MeRIP-qPCR analyses depicted the relative enrichment of circ_104797 when immunoprecipitated with m^6^A antibody (m^6^A-IP) versus immunoglobulin G (IgG) in T24/CDDP (**A**) and 5637/CDDP (**B**) cells in both the presence and absence of ALKBH5 overexpression; **C** expression levels of circ_104797 were assessed using RT-qPCR following ALKBH5 overexpression; **D**, **E** examination of circ_104797 expression in T24/CDDP (**D**) and 5637/CDDP (**E**) cells post actinomycin D treatment considering the presence or absence of ALKBH5 overexpression; **F** a schematic representation highlights the AGACU m^6^A motif situated at the junction of exon 6 and exon 7 in circ_104797; **G** dual-luciferase assays revealed the expression efficiency differences between m^6^A-WT and m^6^A-Mut in BCa cells overexpressing ALKBH5; **H**, **I** RT-qPCR analyses determined the relative abundance of GAPDH mRNA and circ_104797 in T24/CDDP (**H**) and 5637/CDDP (**I**) cells post Act-D treatment, considering the overexpression status of ALKBH5

**Fig. 5:**
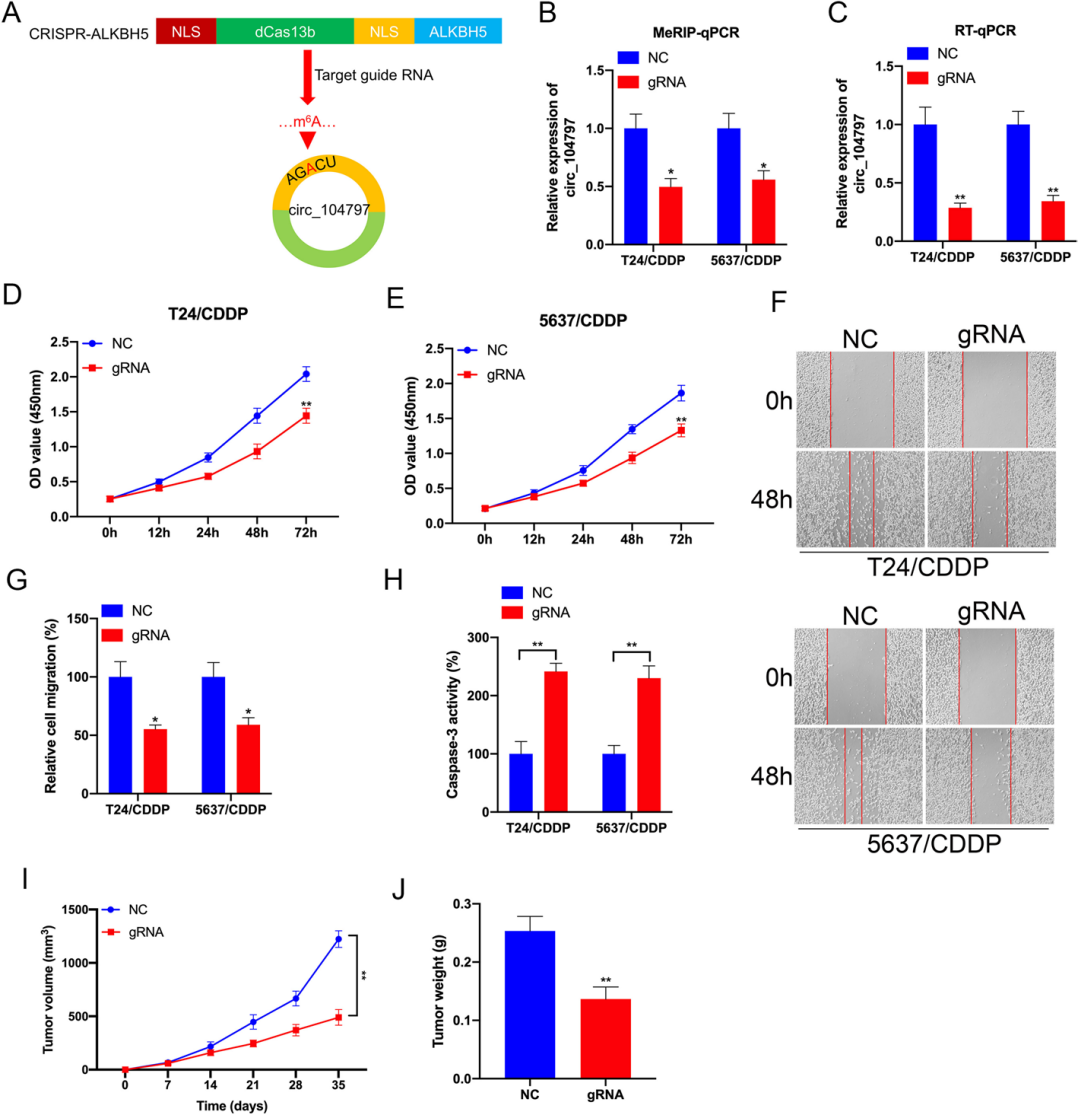
Targeting m^6^A methylation of circ_104797 by CRISPR/dCas13b-ALKBH5 to regulate cisplatin-resistant BCa cells proliferation and apoptosis. **A** A diagrammatic representation delineated the m^6^A site locations within circ_104797 and the regions targeted by specific guide RNA, **B**,**C** In both T24/CDDP and 5637/CDDP cells, m^6^A modifications (**B**) and expression patterns (**C**) of circ_104797 were assessed post-transfection with dCas13b-ALKBH5, either in conjunction with control gRNA or circ_104797-specific gRNA; **D**,**E** the proliferative capacity of T24/CDDP (**D**) and 5637/CDDP (**E**) cells, post-transfection with dCas13b-ALKBH5 and either control gRNA or circ_104797-specific gRNA, was gauged using the CCK-8 assay; **F**,**G** wound healing assays were conducted on T24/CDDP and 5637/CDDP cells post-transfection with dCas13b-ALKBH5 and the respective gRNAs, with representative images (**F**) and subsequent quantitative evaluations (**G**) presented; **H** apoptotic rates in T24/CDDP and 5637/CDDP cells, post-transfection with dCas13b-ALKBH5 and the respective gRNAs, were determined through ELISA; **I**, **J** the tumor volume and weights of BCa cells stably transfected with dCas13b-ALKBH5 combined with gRNA control or gRNA for circ_104797

The intricate m^6^A regulatory network comprises writers (e.g., METTL3 and METTL14, which are m^6^A methyltransferases), erasers (e.g., FTO and ALKBH5, functioning as m^6^A demethylases), and readers (e.g., YTHDF1 and IGF2BP2) that facilitate m^6^A-mediated activities [33]. Our observations revealed a marked reduction in circ_104797 levels immunoprecipitated by the m^6^A antibody in ALKBH5-overexpressing T24/CDDP and 5637/CDDP cells (Fig. 4A-B). Concurrently, qRT-PCR analyses confirmed a significant downregulation of circ_104797 in these ALKBH5-overexpressing cells (Fig. 4C). To assess the influence of ALKBH5 overexpression on circ_104797 stability, we exposed T24/CDDP and 5637/CDDP cells to actinomycin D for varying durations (0, 12, or 24 h). The results indicated diminished stability of linear circ_104797 in the presence of elevated ALKBH5 (Fig. 4D, E). Subsequently, we engineered an expression vector for circ_104797 (termed m^6^A-WT) and introduced mutations at the m^6^A modification site, resulting in m^6^A-Mut (Fig. 5F). Dual-luciferase assays revealed that m^6^A-WT expression was notably suppressed in BCa cells overexpressing ALKBH5 compared with controls. In contrast, m^6^A-Mut expression remained largely unaffected in the presence of elevated ALKBH5 (Fig. 5G). Further, actinomycin D treatments suggested reduced mRNA stability for m^6^A-WT in ALKBH5-overexpressing BCa cells relative to controls (Fig. 5H). However, the m^6^A-Mut variant negated this difference in RNA half-life between control and ALKBH5-overexpressing cells (Fig.5I). Collectively, our findings underscore the role of m^6^A modification in enhancing the stability of circRNA_104797 expression.

### Targeting m^6^A demethylation of circRNA_104797 by CRISPR/dCas13b-ALKBH5 to regulate CDDP-resistant BCa cells proliferation and apoptosis

In our research, we adopted a novel strategy to suppress the m^6^A modification of circRNA_104797. This involved the use of a fusion protein, combining the catalytically inert Cas13b enzyme with the m^6^A demethylase ALKBH5, termed as dCas13b-A5 [34]. We designed specific guide RNAs to guide the dCas13b-A5 complex to precise locations around the m^6^A site within circRNA_104797 (Fig. 5A). Initial validation using MeRIP-qPCR analysis in CDDP-resistant BCa cells confirmed the suppression of m^6^A methylation levels of circRNA_104797 by dCas13b-A5 (Fig. 5B). This was followed by a marked decrease in circRNA_104797 expression when targeted with dCas13b-A5 in T24/CDDP and 5637/CDDP cells (Fig. 5C). To delve deeper into the consequences of dCas13b-A5-mediated intervention on circRNA_104797 in BCa cell dynamics, we assessed cellular proliferation and apoptosis post-transfection with either control or circRNA_104797-specific guide RNA, in tandem with dCas13b-A5, in T24/CDDP and 5637/CDDP BCa cells. The data revealed that targeting circRNA_104797 using its specific gRNA led to a pronounced decline in cell proliferation and migration, while simultaneously enhancing apoptosis, in comparison with cells transfected with non-specific control gRNA and dCas13b-A5 (Fig. 5D–H). Control or gRNA for circRNA_104797 combined with dCas13b-A5 stable BCa cells were used to establish xenografts. Consistently, xenograft model confirmed that m^6^A demethylation of circRNA_104797 can inhibit tumor growth in vivo (Fig. 5I, J).

### circRNA_104797 sustains cisplatin resistance by acting as a miRNA sponge for miR-103a and miR-660-3p

**Fig. 6:**
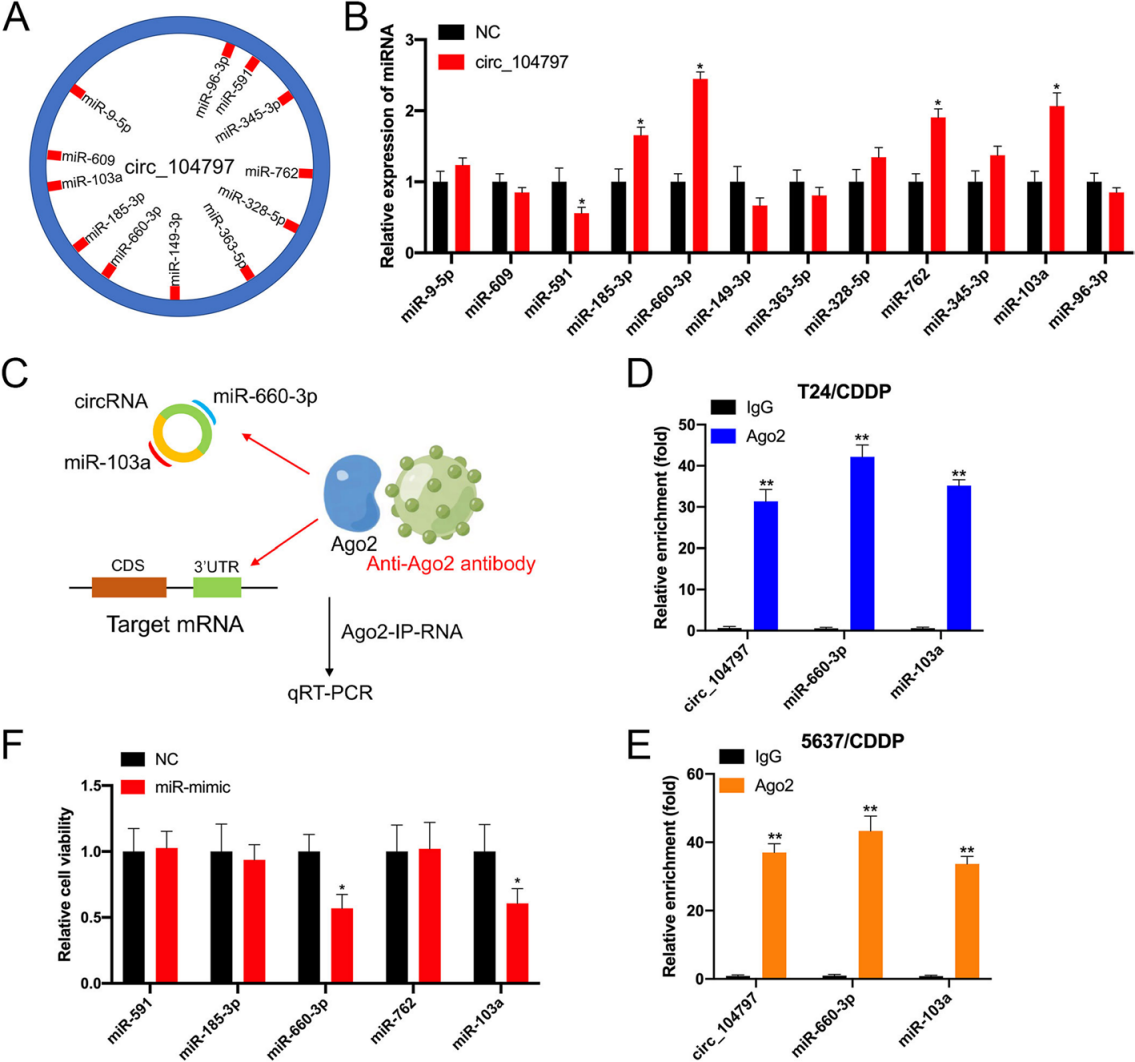
circ_104797 acts as an efficient miRNA sponge for miR-103a and miR-660-3p. **A** A diagrammatic representation highlights the potential binding regions of miRNAs in relation to circ_104797; **B** qRT-PCR results depict the expression levels of the selected miRNAs following the overexpression of circ_104797 in BCa cells; **C** a schematic overview of the Ago2-RIP procedure is provided; **D**, **E** post-Ago2 RIP assay, qRT-PCR was conducted to assess the expression levels of circ_104797, miR-103a, and miR-660-3p in T24/CDDP (**D**) and 5637/CDDP (**E**) cells; **F** CCK-8 assays were performed on cisplatin-resistant BCa cells post-transfection with respective miRNA mimics and control sequences

**Fig. 7:**
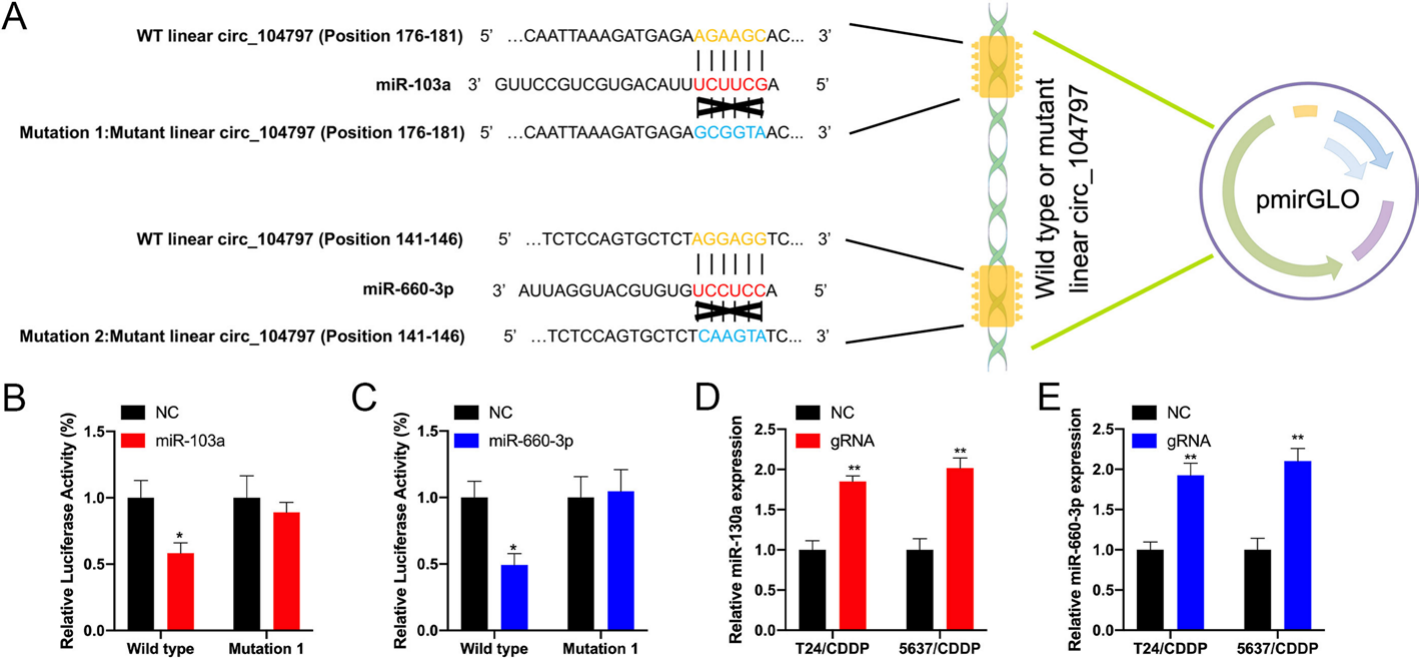
circRNA_104797 sustains cisplatin resistance by acting as a miRNA sponge for miR-103a and miR-660-3p. **A** Diagrammatic representation of the luciferase reporter vectors for wild-type (WT) and mutant (MUT) circ_104797; **B**, **C** luciferase activity measurements for both WT and MUT linear circ_104797 post-transfection with miR-103a (**B**) and miR-660-3p (**C**) mimics in cisplatin-resistant BCa cells; **D**, **E** post-demethylation of circ_104797 via CRISPR/dCas13b-ALKBH5, RT-qPCR was conducted to determine the expression levels of miR-103a and miR-660-3p in T24/CDDP (**D**) and 5637/CDDP (**E**) cells

circRNAs exhibit multifaceted functionalities, encompassing roles such as miRNA sequestration, protein interaction, transcriptional enhancement, and peptide encoding [35]. Given our earlier observation of circRNA_104797’s cytoplasmic localization, we postulated that it might exert its effects through miRNA sponging. To explore potential circRNA-miRNA interactions, we utilized Arraystar’s miRNA target prediction software, which integrates TargetScan [36] and miRanda [37] algorithms (Fig. 6A). Out of the predicted miRNAs, four were identified to interact with circRNA_104797 (Fig. 6B). Subsequent RNA immunoprecipitation (RIP) assays were conducted to isolate RNA molecules interacting with Ago2 in T24/CDDP and 5637/CDDP cells (Fig. 6C). qRT-PCR analyses revealed that circRNA_104797, along with miR-103a and miR-660-3p, were effectively immunoprecipitated by the anti-Ago2 antibody (Fig. 6D, E). Transfection experiments using miRNA mimics indicated that miR-103a and miR-660-3p attenuated cisplatin resistance in BCa cells (Fig. 6F). Based on the predicted miRNA binding domains on circRNA_104797, luciferase reporter assays with both wild-type and mutated linear versions of circRNA_104797 demonstrated that miR-103a and miR-660-3p specifically targeted the wild-type linear variant, leading to reduced Renilla luciferase activity. However, this was not observed with the mutant forms (Fig. 7A–C). Following circRNA_104797 demethylation by dCas13b-A5, the expression of miR-103a and miR-660-3p surged in T24/CDDP and 5637/CDDP cells (Fig. 7D, E). Collectively, these findings underscore the potential of circRNA_104797 to sponge miR-103a and miR-660-3p, influencing cisplatin resistance in BCa.

### circRNA_104797 demethylation inhibited CDDP resistance in BCa cells through targeting miR‑103a and miR-660-3p

**Fig. 8:**
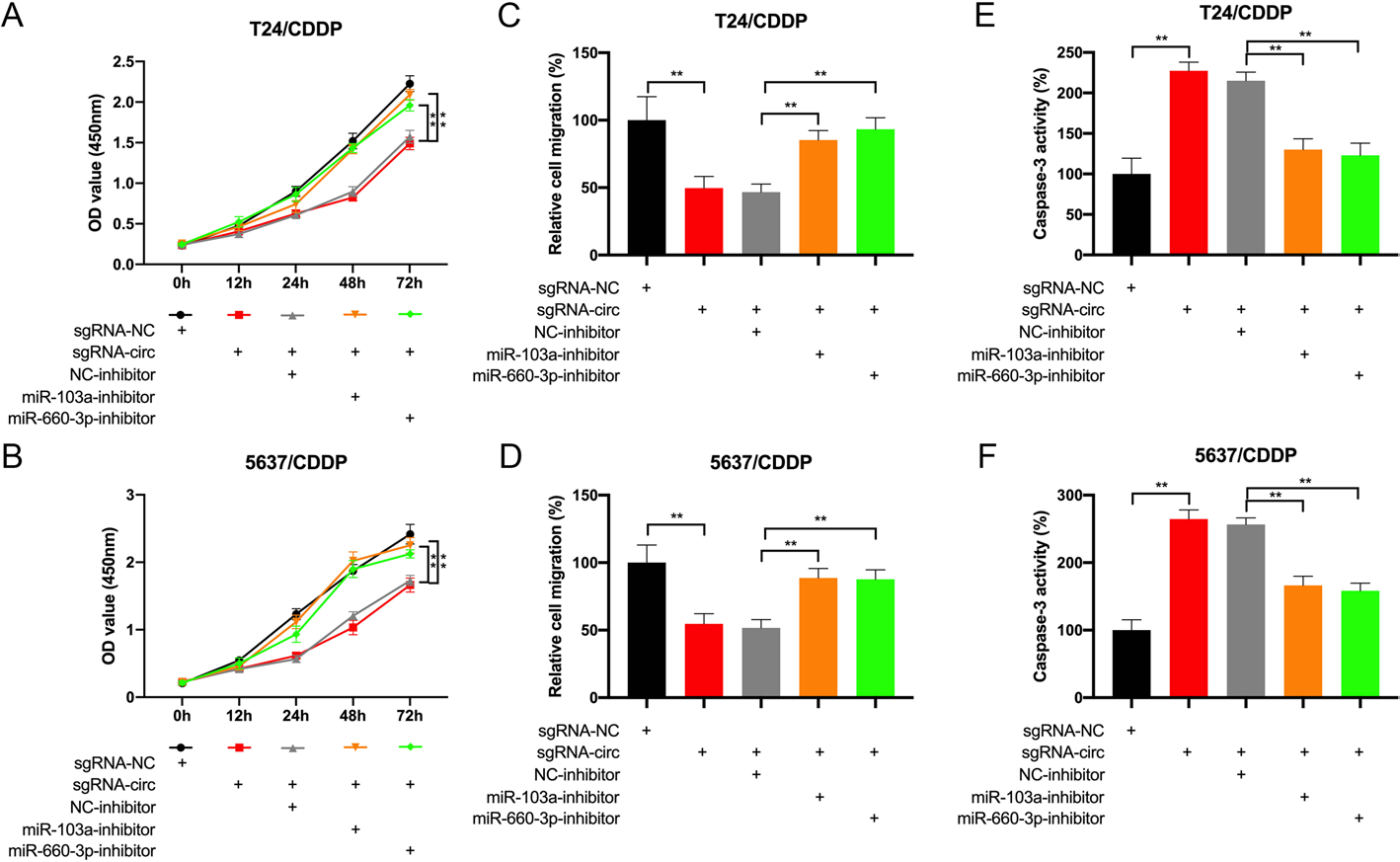
circ_104797 demethylation inhibited CDDP resistance in BCa cells through targeting miR-103a and miR-660-3p. **A**, **B** Proliferation analysis of T24/CDDP (**A**) and 5637/CDDP (**B**) cells following demethylation of circ_104797 and concurrent knockdown of miR-103a and miR-660-3p; **C**, **D** migration evaluation of T24/CDDP (**C**) and 5637/CDDP (**D**) cells post-demethylation of circ_104797 and simultaneous suppression of miR-103a and miR-660-3p; **E**, **F** apoptosis assessment in T24/CDDP (**E**) and 5637/CDDP (**F**) cells after circ_104797 demethylation and concurrent downregulation of miR-103a and miR-660-3p

Having established the circ_104797/miR-103a and miR-660-3p axis in BCa cells, we proceeded to ascertain whether circ_104797 modulated cisplatin resistance in BCa cells by sequestering miR-103a and miR-660-3p. Post-cisplatin treatment of these BCa cells, CCK-8 assay outcomes revealed that the inhibitors of miR-103a and miR-660-3p could partially restore cell viability in T24/CDDP and 5637/CDDP cells subjected to circ_104797 demethylation (Fig. 8A, B). Subsequently, we assessed the influence of miR-103a and miR-660-3p on BCa cell migratory capabilities. Our findings indicated that, while demethylation of circ_104797 curtailed the migratory potential of BCa cells, the concurrent inhibition of miR-103a and miR-660-3p mitigated this suppressive effect (Fig. 8C, D). Additionally, employing the ELISA assay to gauge cell apoptosis, we observed an elevated apoptosis rate in T24/CDDP and 5637/CDDP cells post circ_104797 demethylation. This apoptotic surge, however, was partially offset by the inhibitors of miR-103a and miR-660-3p (Fig. 8E, F). In summation, our data suggest that circ_104797 fosters BCa progression and heightens cisplatin sensitivity by sequestering miR-103a and miR-660-3p, thereby diminishing its tumorigenic impact.

## Discussion

Many individuals with BCa are diagnosed at advanced stages owing to the absence of distinct symptoms in the disease’s early phases. Current therapeutic strategies, including molecular targeted treatments, often fall short in efficacy. This underscores a pressing medical imperative to devise therapies that can extend patient survival. Cisplatin, a molecular targeted drug sanctioned by the US Food and Drug Administration, has been shown to extend the median overall survival time by 3 months in patients with BCa [38]. However, its transient and limited effectiveness, coupled with side effects, such as rashes, diarrhea, hypertension, and hand-foot syndrome, restricts its high-dose administration. The STORM trial further indicated that patients with BCa undergoing aggressive treatments, such as resection or ablation, did not derive significant benefits from adjuvant cisplatin therapy, complicating its clinical utility. The emergence of cisplatin resistance accentuates the necessity for innovative therapeutic solutions. Several mechanisms have been proposed to underlie BCa’s diminished responsiveness to cisplatin, encompassing pathways such as Wnt/β-catenin, TGFβ, Ras/MEK/ERK, PI3K/Akt, TNFα/NF-κB, and JAK/STAT, as well as factors such as autophagy, epithelial–mesenchymal transition, cancer stem cells, tumor microenvironment, and epigenetic alterations [39]. Endeavors to surmount cisplatin resistance and reduce its effective concentration have led to the exploration of combination therapies. However, the overall prognosis for bladder cancer remains suboptimal. In this research, we established cisplatin-resistant cell lines to emulate the resistance observed in patients with BCa.

Over two decades ago, circRNAs were sporadically discovered and initially perceived as rare byproducts arising from transcriptional splicing anomalies. However, with the advent of advanced sequencing techniques and innovative computational methodologies, it became evident that circRNAs, whether derived from exons or introns, are abundant and multifaceted non-coding RNAs (ncRNAs) involved in a plethora of physiological and pathological processes [11, 12]. Contemporary research suggests that circRNAs exert their influence through RNA and protein interactions or by modulating transcription and splicing [35]. For instance, CDR1as, a well-studied circRNA, contains over 60 conserved miR-7 binding sites [12]. Moreover, certain circRNAs, like circHIPK3, have the capacity to interact with multiple miRNAs [19]. Specific examples include hsa_circ_0091570, which acts as a competitive endogenous RNA (ceRNA) for miR-1307, elevating ISM1 expression and thereby influencing hepatocellular cancer progression [40]. Similarly, hsa_circ_0074834 enhances osteogenic differentiation and bone repair by modulating VEGF and ZEB1 expressions via miR-942-5p [41]. Aligning with these findings, our study demonstrated that circ_0058063 sequesters miR-335-5p, leading to an upregulation of B2M in a ceRNA-mediated fashion. Furthermore, the circRNAs/miRNAs axis has been extensively documented in the context of chemo-resistance. For instance, Sang et al. highlighted that circRNA_0025202 curtails tumor growth and tamoxifen sensitivity in breast cancer via the miR-182-5p/FOXO3a pathway [42], while Huang et al. pinpointed circAKT3’s role in augmenting cisplatin resistance in gastric cancer by modulating the miR-198/PI3KR1 axis [43]. In our investigation, we corroborated that circ_0058063 modulates cisplatin resistance in BCa by targeting miR-335-5p. Specifically, we discerned that circ_0058063’s enhancement of cisplatin sensitivity in BCa cells was negated upon miR-335-5p inhibition. Additionally, beta-2-microglobulin (B2M) is an intrinsic low-weight serum protein pivotal for immune responses and angiogenesis. Studies have indicated elevated B2M expression in prostate cancer cells, linking it to metastatic tendencies [44], and surges in serum B2M levels in multiple myeloma patients have been associated with enhanced angiogenesis and myelosuppression [45].

The m^6^A RNA modification, initially identified in the 1970s, has garnered renewed interest owing to advancements in RNA-sequencing methodologies and the elucidation of associated proteins. The m^6^A modification process involves “writers” (including METTL3, METTL14, KIAA1429, WTAP, RBM15, and ZC3H13), “erasers” (such as FTO and ALKBH5), and “readers” (YTHDC1, YTHDC2, YTHDF1, YTHDF2, and HNRNPC) [46]. These modifications are implicated in various RNA functions, including its synthesis, stability, and interactions, particularly in oncological contexts [47]. For instance, the long non-coding RNA (lncRNA) MALAT1, which is conserved and exhibits significant m^6^A methylation, utilizes two m^6^A residues to facilitate an “m^6^A switch” mechanism, enhancing the binding affinity of hnRNPC to a specific region in MALAT1 [48]. Moreover, hnRNPA2B1 can identify pri-miRNAs marked with m^6^A, fostering the interaction between DGR8 and pri-miRNAs, thereby facilitating miRNA processing [49]. In hepatocellular carcinoma (HCC), the implications of m^6^A modifications are multifaceted and remain a subject of debate. While YTHDF2 knockdown has been shown to inhibit HCC cell proliferation [50], other studies have highlighted its tumor-suppressive role by targeting genes such as EGFR, IL11, and SRPINE2 [51]. Recent discoveries have unveiled cell-specific expressions of m^6^A-modified circRNAs [47]. Such modifications can engage YTHDF3 and the initiation factor eIF4G2, modulating protein synthesis from circRNAs [30]. Alterations in m^6^A levels, mediated by ALKBH5 and METTL3, can influence circRNA biogenesis during spermatogenesis by enhancing splicing and fostering circRNA generation [52]. The decay of m^6^A-containing circRNAs is also associated with the YTHDF2–HRSP12–RNase P/MRP-mediated endoribonucleolytic cleavage process [29]. Nevertheless, the roles of m^6^A-modified circRNAs in bladder cancer and their potential influence on cisplatin resistance remain to be fully elucidated.

In our research, we pinpointed a putative m^6^A site within circRNA_104797 through comprehensive experimental analyses. Our findings revealed an elevated m^6^A modification in circRNA_104797 within cisplatin-resistant BCa cells. Furthermore, when the m^6^A modification of circRNA_104797 was curtailed using the CRISPR/dCas13b-ALKBH5 system, its expression notably diminished. Delving into the underlying mechanisms, our study posits that m^6^A modification contributes to the stabilization of circRNA_104797. The precise cause for the augmented m^6^A levels of circRNA_104797 in cisplatin-resistant cells remains an area warranting further exploration. Moreover, our data elucidated that the demethylation of circRNA_104797 curtailed the aggressive traits of BCa cells in cisplatin-resistant scenarios by acting as a sponge for miR-103a and miR-660-3p. This underscores the potential therapeutic value of targeting the circRNA_104797/miR-103a and miR-660-3p axis in mitigating BCa progression, especially in cisplatin-resistant contexts.

Our findings indicate that circ_104797 enhances bladder cancer progression and cisplatin resistance by sequestering miR-103a and miR-660-3p. To elucidate this mechanism further, we analyzed how these microRNAs affect specific cellular pathways and behaviors in bladder cancer cells. MiR-103a and miR-660-3p are known to play critical roles in regulating key oncogenic pathways, such as PI3K/AKT and MAPK/ERK. Their sequestration by circ_104797 leads to the activation of these pathways, resulting in increased cell proliferation, evasion of apoptosis, and enhanced metastatic potential.

Moreover, the interaction of miR-103a and miR-660-3p with circ_104797 appears to modulate the sensitivity of bladder cancer cells to cisplatin. This is particularly evident in their role in regulating drug efflux mechanisms and DNA repair processes. For example, miR-103a is implicated in modulating the expression of genes associated with the cell’s ability to expel chemotherapeutic agents, while miR-660-3p has been linked to the regulation of genes involved in DNA damage response and repair. The dysregulation of these miRNAs by circ_104797 could therefore contribute to the reduced efficacy of cisplatin in bladder cancer treatment.

Our study highlights the pivotal role of circ_104797 in the development and progression of bladder cancer, particularly in the context of cisplatin resistance. The ability of circ_104797 to sponge miR-103a and miR-660-3p, as evidenced in our findings, underscores its contribution to the complex molecular mechanisms driving drug resistance. This not only enhances our understanding of bladder cancer biology but also opens new avenues for therapeutic interventions.

In terms of clinical relevance, our research suggests that targeting circ_104797 could be a promising strategy to overcome the limitations of current treatment approaches, particularly in cisplatin-resistant bladder cancer. For instance, disrupting the interaction between circ_104797 and miRNAs or modulating its m^6^A modification may sensitize cancer cells to chemotherapy, thereby improving treatment efficacy and patient outcomes.

Furthermore, our study paves the way for future research to explore circ_104797 as a potential biomarker for predicting treatment response in bladder cancer. This could lead to more personalized treatment strategies, tailoring therapies based on the circRNA profile of individual tumors.

## Conclusions

To summarize, our research elucidated that the demethylation of circ_104797 augments the sensitivity of BCa cells to cisplatin while concurrently inhibiting their malignant characteristics. Through our findings we underscore the potential of the circ_104797/miR-103a and miR-660-3p regulatory axis as a cornerstone for devising innovative therapeutic interventions aimed at bolstering cisplatin responsiveness in BCa.

## Data Availability

The data that support the findings of this study are available from the corresponding author upon reasonable request.

## Abbreviations

BCa: Bladder cancer
CDDP: Cisplatin
circRNA: Circular RNA
m^6^A: N^6^-methyladenosine
NMIBC: Non-muscle invasive bladder cancer
TURBT: Transurethral resection of the bladder tumor
pre-mRNA: Precursor mRNA
EMT: Epithelial–mesenchymal transition
RIP: RNA immunoprecipitation
NcRNAs: Non-coding RNAs
CeRNA: Competitive endogenous RNA
HCC: Hepatocellular carcinoma
LncRNA: Long non-coding RNA
OS: Overall survival
ATCC: American Type Culture Collection
RT-PCR: Real-time polymerase chain reaction
FISH: Fluorescence in situ hybridization
MeRIP-qPCR: Methylated RNA immunoprecipitation PCR
SD: Standard deviation
ANOVA: One-way analysis of variance
